# Development and content validation of the Childhood Early Oral Aging Syndrome (CEOAS) index for the deciduous dentition: Research protocol

**DOI:** 10.1371/journal.pone.0310543

**Published:** 2024-10-25

**Authors:** Amanda Rafaelly Honório Mandetta, Thais Gimenez, Ana Paula Taboada Sobral, Sandra Gouveia Spinola, Marcela Leticia Leal Gonçalves, Paulo Vinícius Soares, Elaine Marcilio Santos, José Carlos Pettorossi Imparato, Anna Carolina Ratto Tempestini Horliana, Raquel Agnelli Mesquita-Ferrari, Lara Jansiski Motta, Kristianne Porta Santos Fernandes, Sandra Kalil Bussadori

**Affiliations:** 1 Postgraduation Programme in Biophotonic Medicine, Universidade Nove de Julho, São Paulo, Brazil; 2 School of Dentistry, Universidade de São Paulo, São Paulo, Brazil; 3 Faculdade São Leopoldo Mandic, Graduate Program in Pediatric Dentistry, Campinas, SP, Brazil; 4 Postgraduation Programme in Health and Environment, Universidade Metropolitana de Santos, Santos, Brazil; 5 School of Dentistry, Universidade Metropolitana de Santos, Santos, Brazil; 6 Postgraduate Programme in Rehabilitation Sciences, Universidade Nove de Julho, São Paulo, Brazil; 7 Department of Restorative Dentistry, School of Dentistry, Universidade Federal de Uberlândia, Uberlândia, MG, Brazil; Federal University of Minas Gerais: Universidade Federal de Minas Gerais, BRAZIL

## Abstract

Premature, non-physiological tooth wear in childhood has numerous repercussions for oral health. This is a growing problem with multifactorial causes and associated with the current lifestyle. The introduction of new concepts and indices is crucial for enhancing the understanding and management of dental conditions. In this context, we present the Childhood Early Oral Aging Syndrome (CEOAS) and the associated index, which aim to assess early enamel wear in the primary dentition. Rather than focusing solely on isolated etiologies, the index proposes a comprehensive evaluation of signs and symptoms, considering various factors that contribute to the premature and non-physiological loss of dental structure, including wear of both intact and hypomineralized enamel. Indices that evaluate the main factors of early oral aging in childhood and their interactions are fundamental for understanding the condition and developing effective control and treatment strategies, particularly given that there is currently no global index encompassing this information. The aim of the present study was the development and determination of content validity of the Childhood Early Oral Aging Syndrome (CEOAS) index for the primary dentition as a diagnostic and epidemiological survey tool.

**Trial registration**: ClinicalTrials.gov ID: NCT06378385.

## Introduction

The increase in premature, non-physiological tooth wear in childhood has numerous repercussions for oral health. This constitutes a growing phenomenon associated with multifactorial causes and the current lifestyle. With more in-depth knowledge on dental caries and its control, new demands emerge that need to be recognized by healthcare providers to ensure prevention as well as the control of the disease and etiological factors [1].

Non-carious diseases lead to progressive, irreversible dental structure loss, with initial causes unrelated to bacterial involvement [2]. Tooth wear, influenced by age, encompasses erosion, attrition, and abrasion, resulting in varying degrees of structure loss. Pathological tooth wear, marked by unacceptable levels of wear, can cause sensitivity and impact aesthetics and function [3]. The rising prevalence of non-carious oral issues underscores the need for improved practices, health education, research, training, and a transdisciplinary approach [1]. Conditions like erosive wear and enamel defects, including molar-incisor hypomineralization (MIH), hypomineralized second primary molar (HSPM), and dental fluorosis, are a growing concern globally [4].

Recent systematic reviews on erosive wear in children and adolescents faced challenges due to differing indices used for assessment [4]. The Basic Erosive Wear Examination (BEWE), established in 2008, is widely used but focuses solely on erosive etiology, neglecting other contributing factors like attrition and abrasion [5]. Enamel defects, such as hypomineralization, make teeth more prone to wear and accelerate early aging [4].

Enamel defects of higher prevalence have a significant impact on the oral health of affected children [1]. Understanding the current prevalence of early enamel wear in the primary dentition, caused by various etiologies such as abrasion, attrition, and acid erosion, or the combination of these factors, is crucial. Additionally, it is essential to investigate the synergistic effect of these factors on teeth with compromised mineral structure. This knowledge is of paramount importance for the development and direction of public health policies aimed at the prevention and appropriate treatment of these conditions, as well as the promotion of better oral health in the pediatric population. Currently, there is a gap in the literature regarding the precise quantification of the prevalence and the combined etiological factors contributing to early enamel wear in children, highlighting the need for comprehensive epidemiological studies to guide effective public health strategies.

The introduction of new concepts and indices is important for advancing the understanding and management of dental conditions. In this context, we present the Childhood Early Oral Aging Syndrome (CEOAS) and the associated index proposed for validation. This concept aims to unify the assessment of early enamel wear in the primary dentition by relying on observed signs and reported symptoms from patients, rather than focusing solely on isolated etiologies. The index seeks to integrate and evaluate various factors that may contribute to premature and non-physiological loss of dental structure, including both intact and hypomineralized enamel wear. The goal is to provide a more comprehensive and practical approach to diagnosing and treating early wear, considering the complex interaction of the involved factors. Thus, the proposed index may contribute to a more effective approach to identifying and managing early wear in children.

Therefore, the aim of the present study was the development and validation of a Childhood Early Oral Aging Syndrome index for the primary dentition as a diagnostic and epidemiological survey tool.

## Materials and methods

### Study design

The methods will be structured in two distinct steps. For clarity, we follow the recommendations for COSMIN (Consensus-based Standards for the selection of health Measurement Instruments).

1. Development of the index
2. Content validation by specialists
  2.1 Creation of the content validation form
  2.2 Selection of reviewers (specialists)
  2.3 Determination of content validity
  2.4 Review of items
  2.5 Attributing a score to each item
  2.6 Calculation of content validity index (CVI)

### Development of CEOAS index

The aim of the Childhood Early Oral Aging Syndrome index is to investigate clinical signs and symptoms related to the early loss of dental structure associated with the most prevalent enamel defects, which significantly contribute to oral aging. The clinical management of tooth wear is also addressed in CEOAS scores 1, 2 and 3. Current indices used for non-carious conditions do not enable the concomitant investigation of enamel defects, which makes the proposed index innovative and of extreme important to epidemiological surveys. Understanding the prevalence of factors that either separately or synergically accelerate the process of early aging is useful to the establishment of novel treatment strategies.

The CEOAS index involves scores of 0 to 3 for the assessment of tooth wear and dental management, whereas scores of I, II and III are used concomitantly in cases of the presence of enamel defects, as shown in (Table 1).

**Table 1:** Childhood Early Oral Aging Syndrome (CEOAS) index.

**CEOAS index**	
**CEOAS 0**	**Absence of signs**
**CEOAS 1**	**Mild:** Presence of clinical signs in reversible stages, without sensitivity. Such cases require follow-up.
**CEOAS 2**	**Moderate:** Presence of advanced signs with sensitivity and compromised function. Such cases require restorative treatment and management of the sensitivity.
**CEOAS 3**	**Severe:** Presence of severe signs with pulp involvement and the risk of the loss of the tooth. Such cases require more invasive and rehabilitative treatment.
**CEOAS I**	**Presence of PSMH–Demarcated opacities**
**CEOAS II**	**Presence of PSMH–Post-eruptive breakdown (PEB)**
**CEOAS III**	**Presence of fluorosis**

**CEOAS 0:** Absence of signs and symptoms of CEOAS.

**CEOAS 1**: Clinical findings compatible with chronological age and oral age. First clinical signs (facets with initial wear on enamel level without altering function), without symptoms. Such cases require clinical follow-up.

Due to the lack of studies on the physiological wear pattern in the primary dentition, slight tooth wear without symptoms, functional or esthetic problems compatible with physiological wear is considered in this score.

**CEOAS 2**: Signs of wear not compatible with chronological age (facets with deep wear, with dentin exposure and compromised function) and symptoms of hypersensitivity. May have gingival recession. Such cases require restorative treatment and management of the sensitivity. Due to the lack of studies on the physiological wear pattern in the primary dentition, atypical tooth wear for the age of the patient (pathological wear), with symptoms as well as functional and esthetic problems are considered in this score.

**CEOAS 3**: Signs of severe wear not compatible with chronological age, with pulp involvement (inflammation or necrosis), compromising function and the stomatognathic system. May have tooth fissures, root fissures, tooth fractures, gingival recession and changes in the temporomandibular joint. Loss of the tooth may occur. Such cases require invasive treatment (endodontic, restorative, rehabilitative or extractive).

**CEOAS I:** In the presence of primary second molar hypomineralization (PSMH) with demarcated opacities and without post-eruptive breakdown (PEB), the CEOAS I score should be recorded concomitantly with the 1, 2 or 3 score detected in the clinical examination.

**CEOAS II:** In the presence of primary second molar hypomineralization (PSMH) with post-eruptive breakdown (PEB), the CEOAS II score should be recorded concomitantly with the 1, 2 or 3 score detected in the clinical examination.

**CEOAS III:** In the presence of dental fluorosis, the CEOAS III score should be recorded concomitantly with the 1, 2 or 3 score detected in the clinical examination.

**Fig 1:**
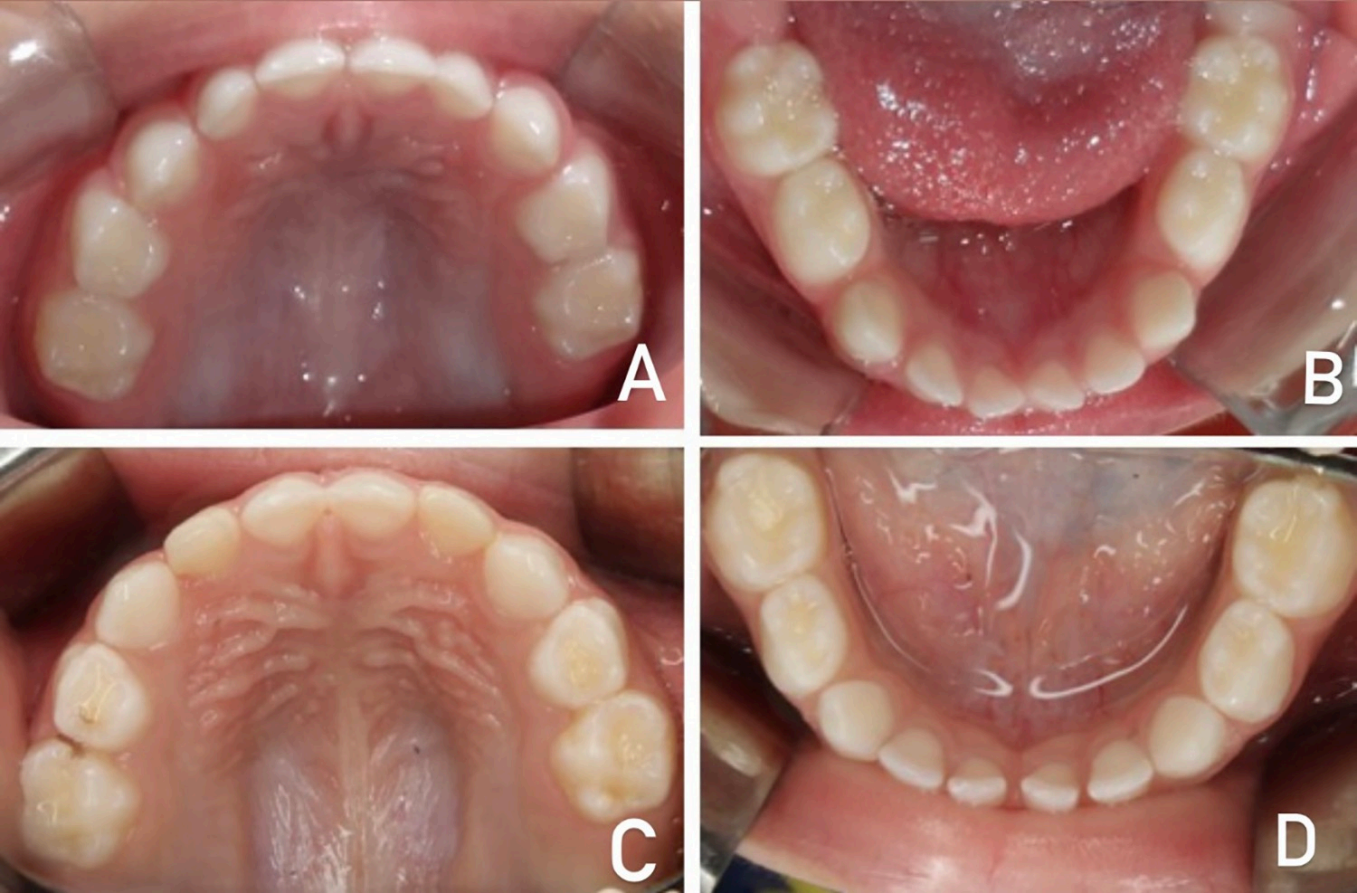
CEOAS 0 (A and B) and CEOAS 1 (C and D) scores.

**Fig 2:**
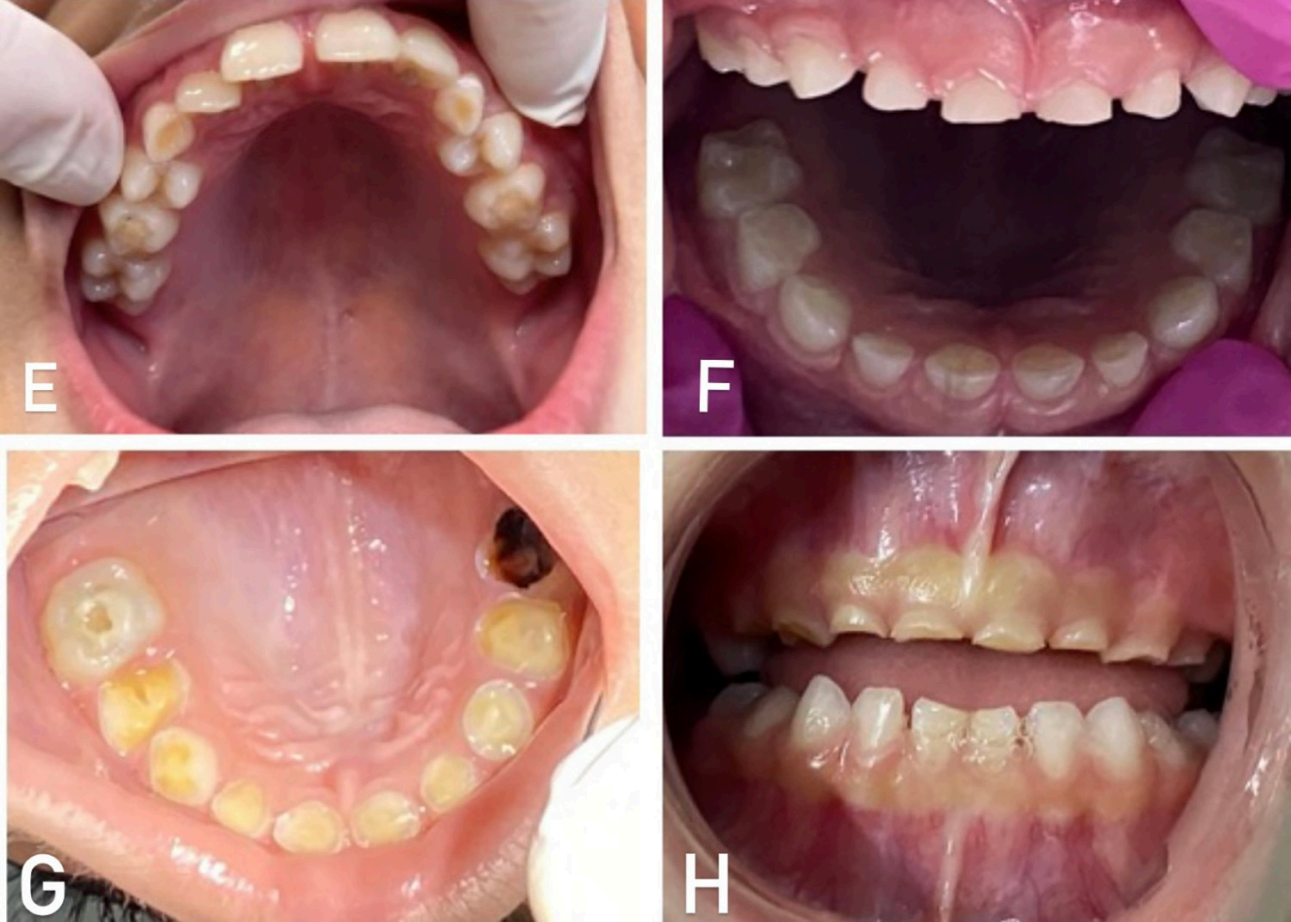
CEOAS 2 (E and F) and CEOAS 3 (G and H) scores.

**Fig 3:**
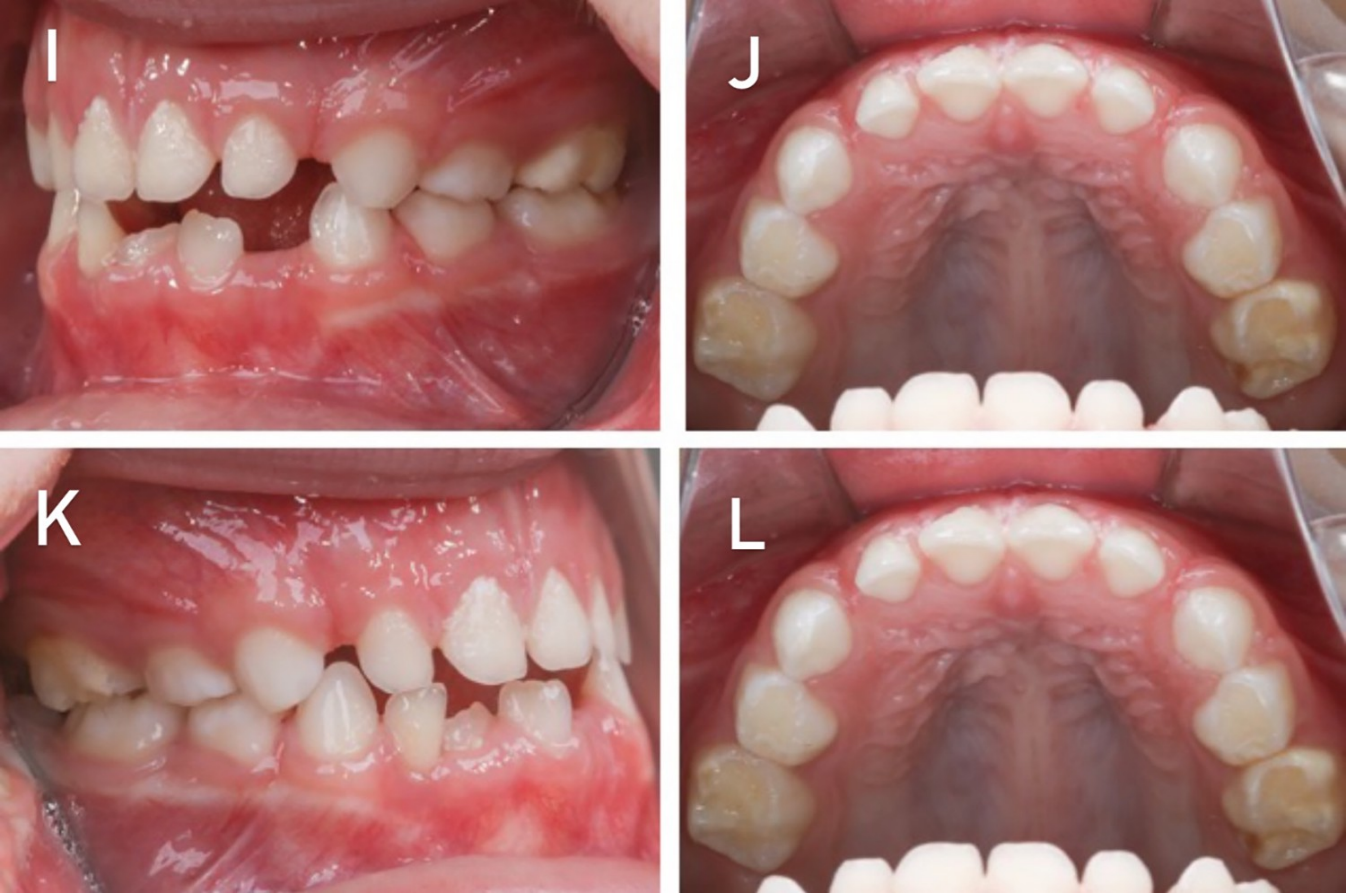
CEOAS 1 associated with CEOAS I (I and J) and CEOAS 1 associated with CEOAS II (K and L) scores.

Images exemplifying the index scores are presented below. In Fig 1, CEOAS 0 (A and B) and CEOAS 1 (C and D) scores are presented. In Fig 2, CEOAS 2 (E and F) and CEOAS 3 (G and H) scores are presented. In Fig 3, CEOAS 1 associated with CEOAS I (I and J) and CEOAS 1 associated with CEOAS II (K and L) scores are presented. In images I and J, enamel defects of the type Hypomineralization of Second Primary Molars (HSPM) without post-eruptive breakdown (PEB) in tooth 65 can be observed. In images K and L, enamel defects of the type Hypomineralization of Second Primary Molars (HSPM) with post-eruptive breakdown (PEB) in tooth 55 can be observed.

The CEOAS index can be used in the simplified version with the score of the greatest severity found. In the complete version, the CEOAS index can be used followed by an odontogram on which each tooth is assigned a score (Table 2).

**Table 2:** Odontogram for Childhood Early Oral Aging Syndrome (CEOAS) index.

Odontogram										
Tooth	55	54	53	52	51	61	62	63	64	65
Tooth	85	84	83	82	81	71	72	73	74	75

### Content validation by specialists

**Creation of content validity form**

**Table:** 

**Content validity of Childhood Early Oral Aging Syndrome (CEOAS) index.** Dear specialists, This index was developed to provide a reliable, standardized tool for the investigation of clinical signs and symptoms related to early oral aging in childhood, with the inclusion of enamel defects, which are currently highly prevalent. The main purpose of the index is to serve as a tool for epidemiological surveys and enable knowledge on the prevalence and severity of tooth wear in the pediatric population, contributing to the planning of health actions based on the data collected. We need your specialized opinion on the degree of relevance of each item to the domains measured. Your review should be based on the relevance, clarity and applicability of the score developed for the CEOAS index, as found in Table 3. We would be grateful for verbal or written suggestions that enable us to improve the content of the index.

#### Selection of reviewers (specialists)

The selection of specialists for the assessment will be based on the individual experience of doctoral professors specializing in pediatric dentistry, who will be chosen to ensure content validity, following recommendations. Following recommendations for content validation, at least six specialists will be selected [6]. The recruitment period for specialists will take place between July 29, 2024, and September 16, 2024.

#### Determination of content validity

Content validation will not be performed in person. The online content validation form will be sent to the specialists with the items and criteria to be assessed (Table 3). A deadline for evaluation will be requested, and the entire process will be monitored. The images used to validate the Early Childhood Oral Aging Syndrome index for deciduous dentition were collected at the Dental Clinic of the Metropolitan University of Santos (UNIMES), in the Pediatric Dentistry discipline and in the Pediatric Dentistry Specialization program between April 1, 2024, and May 31, 2024. After obtaining written consent from parents or guardians, children were also informed, in appropriate language, about the objectives and procedures of the research and agreed to participate voluntarily, expressing their assent, which was recorded in the Informed Assent Form. The protocol for this study was approved by the Human Research Ethics Committee of the Metropolitan University of Santos-UNIMES (certificate number: 77036723.7.0000.5509) and the protocol registered in clinical trials (S1 File). The original research project submitted to the Research Ethics Committee, in both the original language and in English, can be found in the (S2 and S3 Files), respectively.

**Table 3:** Items and assessment and validation criteria of the CEOAS index.

**Items and assessment and validation criteria of the CEOAS index**				
Is the CEOAS index relevant to the diagnosis of early oral aging syndrome?	1	2	3	4
	It is not relevant.	Relevant, but needs major revision.	Relevant, but needs minor revision.	Very relevant
Is the CEOAS index clear for the diagnosis of early oral aging syndrome?	1	2	3	4
	It is not clear	Clear, but needs major revision.	Clear, but needs minor revision.	Very Clear
Is the CEOAS index relevant with regards to communication among health professionals and researchers?	1	2	3	4
	It is not relevant.	Relevant, but needs major revision.	Relevant, but needs minor revision.	Very relevant
Is the CEOAS index applicable for diagnoses and epidemiological surveys?	1	2	3	4
	It is not applicable.	Applicable, but needs major revision.	Applicable, but needs minor revision.	Very applicable

#### Review of items

During the validation process, the specialists will be asked to critically review the content prior to assigning scores. Verbal or written suggests for improving the content will be encouraged.

#### Attributing a score to each item

After the review, the specialists will attribute a score to each item (Table 3) corresponding to their assessments in terms of the relevance, clarity and applicability of the content and send their assessments before the deadline stipulated for the process. To assess the content, if a specialist indicates "1" on Table 3 (Not relevant/Not clear), a discussion meeting will be held with the group of specialists. This meeting will address and clarify any doubts or issues raised, ensuring consensus and accuracy in the content evaluation.

#### Calculation of content validity index (CVI)

The CVI will be calculated on a scale level based on the universal agreement method (S-CVI/UA). The classification of relevance should be recoded as 1 (scale score of 3 or 4) or 0 (scale score of 1 or 2). The universal agreement (UA) score is given as 1 when the item achieves 100% agreement among the specialists (only scores 3 and 4 for all items). Otherwise, the UA score is given as 0. The S-CVI/UA is equal to the sum of the UA scores divided by the number of items.

Sum of UA scores

S-CVI/UA = ______________________

Number of items

CVI values higher than 0.83 will be considered acceptable [7].

After the validation of the index, an observational study will be conducted to identify the prevalence of early wear in the primary dentition using the Childhood Early Oral Aging Syndrome index (CEOAS), and to investigate possible associated etiological factors. The study report will adhere to the STROBE guidelines for cross-sectional studies.

## Supporting information

S1 File ClinicalTrials.gov protocol registration. (PDF)

S2 File Original language research project. (DOCX)

S3 File English research project. (DOCX)
