# Supplementary material for: Development and content validation of the Childhood Early Oral Aging Syndrome (CEOAS) index for the deciduous dentition: Research protocol
Source: PLoS One. 2024 Oct 25;19(10):e0310543. doi: 10.1371/journal.pone.0310543 (PMC11508467; doi:10.1371/journal.pone.0310543)
Supplement: S1 File — (PDF) [file pone.0310543.s001.pdf]

ClinicalTrials.gov PRS
Protocol Registration and Results System

ClinicalTrials.gov Protocol Registration and Results System (PRS) Receipt
Release Date: April 23, 2024

ClinicalTrials.gov ID: NCT06378385

Study Identification

Unique Protocol ID: DevelopmentCEOAS

Brief Title: Development and Content Validation of the Childhood Early Oral Aging Syndrome (CEOAS) Index for the Deciduous Dentition

Official Title: Development and Content Validation of the Childhood Early Oral Aging Syndrome (CEOAS) Index for the Deciduous Dentition

Secondary IDs:

Study Status

Record Verification: April 2024

Overall Status: Not yet recruiting

Study Start: May 1, 2024 [Anticipated]

Primary Completion: October 31, 2024 [Anticipated]

Study Completion: December 20, 2024 [Anticipated]

Sponsor/Collaborators

Sponsor: University of Nove de Julho

Responsible Party: Principal Investigator
Investigator: Sandra Kalil Bussadori [sbussadori]
Official Title: Principal Investigator
Affiliation: University of Nove de Julho

Collaborators:

Oversight

U.S. FDA-regulated Drug:

U.S. FDA-regulated Device:

U.S. FDA IND/IDE:

Human Subjects Review: Board Status: Approved
Approval Number: 77036723.7.0000.5509
Board Name: Universidade Metropolitana de Santos Ethics Committee
Board Affiliation: Universidade Metropolitana de Santos
Phone: +55 13 3228-3400
Email: fernanda.agnelli@unimes.br
Address:

	Av Conselheiro Nébias 536 - Encruzilhada - CEP: 11.045-002 - Santos/SP - Brazil
Data Monitoring:	

Study Description

Brief Summary:	Premature, non-physiological tooth wear in childhood has numerous repercussions for oral health. This is a growing problem with multifactorial causes and associated with the current lifestyle. The aim of the present study was the development and determination of content validity of the Childhood Early Oral Aging Syndrome (CEOAS) index for the primary dentition as a diagnostic and epidemiological survey tool considering the current changes found in this population.
Detailed Description:	

Conditions

Conditions:	Aging
Keywords:	

Study Design

Study Type:	Observational
Observational Study Model:	Case-Only
Time Perspective:	Retrospective
Biospecimen Retention:	None Retained
Biospecimen Description:	
Enrollment:	6 [Anticipated]
Number of Groups/Cohorts:	1

Groups and Interventions

Groups/Cohorts	Interventions
Observational<br>Development and validation of a Childhood Early Oral Aging Syndrome index for the primary dentition as a diagnostic and epidemiological survey tool considering the current changes found in the pediatric population.	Behavioral: Childhood Early Oral Aging Syndrome index<br>Development and validation of a Childhood Early Oral Aging Syndrome index for the primary dentition as a diagnostic and epidemiological survey tool considering the current changes found in the pediatric population.

Outcome Measures

Primary Outcome Measure:

1. Development of CEOAS index
   The Childhood Early Oral Aging Syndrome index will be to investigate clinical signs and symptoms related to the early loss of dental structure associated with the most prevalent enamel defects, which significantly contribute to oral aging. The clinical management of tooth wear is also addressed in CEOAS scores 1, 2 and 3. Current indices used

for non-carious conditions do not enable the concomitant investigation of enamel defects, which makes the proposed index innovative and of extreme important to epidemiological surveys. Understanding the prevalence of factors that either separately or synergically accelerate the process of early aging is useful to the establishment of novel treatment strategies.

The CEOAS index involves scores of 0 to 3 for the assessment of tooth wear and dental management, whereas scores of I, II and III are used concomitantly in cases of the presence of enamel defects, as show in Table 1

[Time Frame: Immediately]

Secondary Outcome Measure:

2. Validation of CEOAS index
   Content validation will not be performed in person. The online content validation form will be sent to the specialists with the items and criteria to be assessed (Table 1) CEOAS 0 Absence of signs CEOAS 1 Mild: Presence of clinical signs in reversible stages, without sensitivity. Such cases require follow-up.

   CEOAS 2 Moderate: Presence of advanced signs with sensitivity and compromised function. Such cases require restorative treatment and management of the sensitivity.

   CEOAS 3 Severe: Presence of severe signs with pulp involvement and the risk of the loss of the tooth. Such cases require more invasive and rehabilitative treatment.

   CEOAS I Presence of PSMH – Demarcated opacities CEOAS II Presence of PSMH – Post-eruption fractures CEOAS III Presence of fluorosis

   [Time Frame: Immediately]

Eligibility

Study Population: Dental surgeons with experience in the subject of Childhood Early Oral Aging Syndrome

Sampling Method: Probability Sample

Minimum Age: 18 Years

Maximum Age: 70 Years

Sex: All

Gender Based:

Accepts Healthy Volunteers: No

Criteria: Inclusion Criteria:

-Dental surgeons

Exclusion Criteria:

-Other healthcare professionals

Contacts/Locations

Central Contact Person:

Central Contact Backup:

Study Officials:

Locations: **Brazil**
Universidade Metropolitana de Santos - UNIMES
Santos, São Paulo, Brazil, 11045-002
Contact: Sandra Kalil sandra.skb@gmail.com

IPDSharing

Plan to Share IPD:

References

Citations:

Links:

Available IPD/Information:

U.S. National Library of Medicine | U.S. National Institutes of Health | U.S. Department of Health & Human Services
