# Supplementary material for: Development and content validation of the Childhood Early Oral Aging Syndrome (CEOAS) index for the deciduous dentition: Research protocol
Source: PLoS One. 2024 Oct 25;19(10):e0310543. doi: 10.1371/journal.pone.0310543 (PMC11508467; doi:10.1371/journal.pone.0310543)
Supplement: S2 File — (DOCX) [file pone.0310543.s002.docx]

**Desenvolvimento e validação de conteúdo do índice da Síndrome do envelhecimento Precoce Bucal Infantil (SEPBI) para dentição decídua.**

**Pesquisadores Responsáveis pelo projeto:**

Amanda Rafaelly Honório Mandetta

São Paulo

2024

**Resumo**

O desgaste dentário precoce e não fisiológico encontrado na idade pediátrica, e com inúmeras repercussões à saúde bucal, é uma realidade crescente, associada a causas multifatoriais e ao estilo de vida atual. O objetivo deste estudo é o desenvolvimento e validação de conteúdo do índice Síndrome do Envelhecimento Precoce Bucal Infantil (SEPBI) para a dentição decídua, como instrumento diagnóstico e de levantamento epidemiológico, considerando as alterações atuais encontradas nesta população.

**Introdução**

O aumento do desgaste dentário precoce e não fisiológico encontrado na idade pediátrica, e com inúmeras repercussões à saúde bucal, é uma realidade crescente, associada a causas multifatoriais e ao estilo de vida atual. Com o conhecimento mais aprofundado sobre a doença cárie e seu controle, novas demandas surgem, com a necessidade de reconhecimento entre os profissionais da saúde, visando a prevenção, controle da doença e dos fatores etiológicos ^1^.

As doenças não cariosas, provocam perda progressiva e irreversível da estrutura dentária, seu processo inicial está associado a causas diversas sem envolvimento bacteriano ^2^.

O desgaste dentário é um fenômeno relacionado à idade. Os desafios erosivos, e de atrição e abrasão ao longo da vida resultarão em graus variáveis de perda de estrutura. É difícil definir e quantificar desgaste patológico, mas o termo patológico tem sido utilizado para descrever níveis inaceitáveis de desgaste progressivo, que podem resultar em sensibilidade, comprometimento estético e problemas funcionais ^3^.

O aumento da prevalência mundial das lesões não cariosas, reflete a necessidade de mudanças de conduta, educação em saúde, levantamento epidemiológico, treinamentos e calibrações, assim como uma perspectiva transdisciplinar ^1^.

Condições odontológicas não cariosas, como desgaste dentário erosivo, defeitos de desenvolvimento de esmalte (DDE), incluindo Hipomineralização de Molares e Incisivos (HMI) Hipomineralização de Segundos Molares Decíduos (HSMD) e Fluorose dentária são objetos de extensa pesquisa e crescente preocupação de dentistas em todo o mundo ^4^.

Estudos epidemiológicos, com duas recentes revisões sistemáticas tentaram avaliar a prevalência dos desgastes erosivos em crianças e adolescentes, no entanto a comparação entre os estudos foi desafiadora, uma vez que diferentes índices foram utilizados para avaliar a gravidade dos desgastes ^4^.

O índice Exame Básico de Desgaste Erosivo Dentário (BEWE) foi elaborado como parte de uma reunião de consenso em 2008 e atualmente é um dos índices mais utilizados em estudos clínicos e epidemiológicos ^5^. No entanto ele leva em consideração apenas a etiologia erosiva como diagnóstico. Embora a erosão seja considerada como a principal causa dos desgastes dentários em crianças, outros fatores podem contribuir de maneira sinérgica, como a atrição e abrasão^2^.

Os defeitos de esmalte do tipo hipomineralizado são altamente prevalentes, e devido a fragilidade do esmalte alterado são mais susceptíveis ao desgaste e perda precoce, contribuindo dessa forma para a aceleração do envelhecimento precoce^4^. Os índices mais utilizados para a fluorose, são Dean e Thylstrup e Fejerskov. Os índices utilizados para a HMI e HSMD são DDE modificado e o índice EAPD^1^.

Atualmente, a ausência de um índice que contemple os principais fatores que determinam a síndrome do envelhecimento precoce infantil e avalie possíveis associações que coexistam e que impliquem no controle e tratamento da doença são necessários e indispensáveis.

Portanto, este estudo tem como objetivo o desenvolvimento e validação de conteúdo de um índice sobre a Síndrome do Envelhecimento Precoce Bucal Infantil para a dentição decídua, como instrumento diagnóstico e de levantamento epidemiológico, considerando as alterações atuais encontradas na população pediátrica.

**Materiais e Métodos**

**Desenho do estudo**

Para melhor compreensão a metodologia será dividida em duas etapas, sendo a primeira etapa direcionada ao desenvolvimento do índice e validação do conteúdo.

1. Desenvolvimento do índice
2. Validação do conteúdo por especialistas
   1. Preparação do formulário de validação do conteúdo
   2. Seleção de revisores especialistas
   3. Realização da validação de conteúdo
   4. Revisão de itens
   5. Fornecer pontuação de cada item
   6. Cálculo do CVI
3. **Desenvolvimento do Índice SEPBI**

O índice da Síndrome do Envelhecimento Precoce Bucal Infantil tem como objetivo avaliar sinais e sintomas clínicos relacionados a perda precoce da estrutura dental associado aos defeitos de esmalte mais prevalentes na atualidade, e que contribuem de maneira significativa para o envelhecimento bucal. O manejo clínico dos desgastes também é abordado nos escores SEPBI 1, 2 e 3. Atualmente os índices utilizados para lesões não cariosas, não abordam de maneira concomitante os defeitos de esmalte, o que torna esse índice inovador e de extrema importância para levantamento epidemiológico. O entendimento de prevalência dos fatores que agem isoladamente ou sinergicamente acelerando o processo do envelhecimento precoce é útil para novas estratégias clínicas de tratamento.

O índice da Síndrome do Envelhecimento Precoce Bucal Infantil (SEPBI) utiliza os escores de 0 a 3 para avaliar o desgaste dentário e manejo odontológico, e escores I, II e III que devem ser utilizados concomitantemente em casos da presença de defeitos de esmalte, conforme Tabela 1:

| **Tabela 1: Índice da Síndrome do Envelhecimento Precoce Bucal Infantil (SEPBI)** | |
| --- | --- |
| **SEPBI 0** | **Ausência de sinais** |
| **SEPBI 1** | **Leve:** Presença de sinais clínicos em estágios reversíveis sem sensibilidade. Necessitam de acompanhamento |
| **SEPBI 2** | **Moderado:** Presença de sinais avançados, com sensibilidade e comprometimento da função. Necessitam de tratamento restaurador e manejo da sensibilidade |
| **SEPBI 3** | **Severo:** Presença de sinais severos com comprometimento pulpar e risco de perda do elemento dental. Necessitam de tratamento mais invasivo e reabilitador |
| **SEPBI I** | **Presença HSMD - Opacidades demarcadas** |
| **SEPBI II** | **Presença HSMD – Fraturas pós eruptivas** |
| **SEPBI III** | **Presença de Fluorose** |

**O SEPBI 0:** Ausência de sinais e sintomas da SEPBI.

**O SEPBI 1**: Achados clínicos ainda compatíveis entre idade cronológica (IC) e idade bucal (IB). Primeiros sinais clínicos (facetas de desgastes iniciais em nível de esmalte e sem alterar função), sem sintomas. Necessário acompanhamento clínico.

Devido a falta de estudos sobre padrão de desgaste fisiológico na dentição decídua, serão considerados nesse escore pequenos desgastes dentários, sem sintomatologia, problemas funcionais e estéticos, compatíveis com o desgaste fisiológico.

**O SEPBI 2**: Sinais de desgaste não compatíveis com a idade cronológica (facetas de desgaste profundas, com exposição de dentina e comprometimento da função), sintomas de hipersensibilidade presentes. Pode apresentar recessão gengival. Necessário tratamento restaurador e de manejo da sensibilidade. Devido a falta de estudos sobre padrão de desgaste fisiológico na dentição decídua, serão considerados nesse escore, desgastes dentários atípicos para a idade do paciente (desgaste patológico), com sintomatologia, problemas funcionais e estéticos.

**O SEPBI 3**: Sinais severos de desgaste, não compatíveis com a idade cronológica, com comprometimento pulpar (inflamação ou necrose), comprometimento da função e do sistema estomatognático. Pode conter trincas dentárias, trincas de raiz, fraturas dentárias, recessão gengival e alterações na articulação temporomandibular (ATM). Pode ocorrer perda do elemento dental. Necessário tratamento invasivo (endodôntia, restaurador, reabilitador e /ou exodontia).

**O SEPBI I:** Na presença de Hipomineralização de Segundos Molares Decíduos (HSMD) com opacidades demarcadas e sem fraturas pós-eruptivas o escore SEPBI I deve ser anotado simultaneamente ao escore 1,2 ou 3 detectado no exame clínico.

**O SEPBI II:** Na presença de Hipomineralização de Segundos Molares Decíduos (HSMD) com fraturas pós-eruptivas o escore SEPBI II deve ser anotado simultaneamente ao escore 0,1,2 ou 3 detectado no exame clínico.

**O SEPBI III:** Na presença de fluorose dentária o escore SEPBI III deve ser anotado simultaneamente ao escore 0,1,2 ou 3 detectado no exame clínico.

O índice SEPBI pode ser utilizado na versão simplificada, com o escore de maior gravidade encontrada. Na versão completa, o SEPBI pode ser utilizado seguido de um odontograma, e cada escore será atribuído a um dente (Figura 1).

| **Figura 1: Odontograma para índice da Síndrome do Envelhecimento Precoce Bucal Infantil (SEPBI)** | | | | | | | | | | |
| --- | --- | --- | --- | --- | --- | --- | --- | --- | --- | --- |
| Dente | 55 | 54 | 53 | 52 | 51 | 61 | 62 | 63 | 64 | 65 |
|  |  |  |  |  |  |  |  |  |  |  |
| Dente | 85 | 84 | 83 | 82 | 81 | 71 | 72 | 73 | 74 | 75 |
|  |  |  |  |  |  |  |  |  |  |  |

1. **Validação do conteúdo por especialistas**

**2.1 Preparação do formulário de validação do conteúdo**

| **Validação de conteúdo do índice da Síndrome do Envelhecimento Precoce Bucal Infantil (SEPBI).**  Prezados especialistas,  Este índice foi desenvolvido com o objetivo de fornecer uma ferramenta confiável e padronizada sobre sinais e sintomas clínicos relacionados ao envelhecimento precoce bucal infantil, com a inclusão dos defeitos de esmalte com alta prevalência na atualidade. O índice tem como principal finalidade o levantamento epidemiológico, para o conhecimento da prevalência e severidade dos desgastes na população pediátrica, proporcionando a partir dos dados coletados, a avaliação e o planejamento de ações de saúde.  Precisamos de sua opinião especializada sobre o grau de relevância de cada item para os domínios medidos. Sua revisão deve ser baseada na relevância, clareza e aplicabilidade dos escores desenvolvidos para o índice SEPBI, conforme formulário encontrado na Tabela 2. Solicitamos que sugestões verbais ou escritas, que forneçam melhora do conteúdo sejam realizadas. |
| --- |

| **Tabela 2: Itens e critérios de avaliação e validação do índice SEPBI** | | | | |
| --- | --- | --- | --- | --- |
| O índice SEPBI é relevante para o diagnóstico da síndrome do envelhecimento bucal? | 1 | 2 | 3 | 4 |
|  | Não é relevante | Relevante, mas precisa de grandes revisões | Relevante, mas precisa de pequenas revisões | Muito relevante |
| O índice SEPBI é claro para o diagnóstico da síndrome do envelhecimento bucal? | 1 | 2 | 3 | 4 |
|  | Não está claro | Claro, mas precisa de grandes revisões | Claro, mas precisa de pequenas revisões | Muito claro |
| O índice SEPBI é relevante no que diz respeito à comunicação entre profissionais e pesquisadores? | 1 | 2 | 3 | 4 |
|  | Não é relevante | Relevante, mas precisa de grandes revisões | Relevante, mas precisa de pequenas revisões | Muito relevante |
| O índice SEPBI é aplicável para diagnóstico e levantamento epidemiológico? | 1 | 2 | 3 | 4 |
|  | Não é aplicável | Aplicável, mas precisa de grandes revisões | Aplicável, mas precisa de pequenas revisões | Muito Aplicável |

**2.2 Seleção de revisores especialistas**

A seleção dos especialistas para avaliação será baseada na experiência individual do profissional no tema abordado neste estudo. Seguindo as recomendações para validação de conteúdo, serão selecionados no mínimo seis profissionais especialistas^6^.

- 1. **Realização da validação do conteúdo**

A validação de conteúdos será realizada de modo não presencial. O formulário de validação de conteúdo online será enviado aos especialistas com os itens e critérios que deverão ser avaliados (Tabela 1). Será solicitado um prazo para a avaliação e todo o processo será acompanhado. As imagens utilizadas para validação do índice da Síndrome do Envelhecimento Precoce Bucal Infantil para a dentição decídua serão coletadas na Clínica da Faculdade de Odontologia da Universidade Metropolitana de Santos (UNIMES) na disciplina de Clínica Infantil e na Especialização de Odontopediatria. Após o consentimento dos pais ou responsáveis, as crianças serão também informadas, em linguagem apropriada, sobre os objetivos e procedimentos da pesquisa e devem concordar em participar voluntariamente da mesma, manifestando seu assentimento, que será registrado no Termo de Assentimento Livre e Esclarecido.

**2.4 Revisão de itens**

Durante o processo de validação os especialistas serão solicitados a revisar criticamente o conteúdo antes de realizar a pontuação. Sugestões verbais e por escrito para melhora do conteúdo serão estimuladas.

- 1. **Fornecer pontuação de cada item**

Após a revisão, os especialistas devem pontuar em cada item (Tabela 2) sua avaliação sobre a relevância, clareza e aplicabilidade do conteúdo e entregá-las dentro do prazo fornecido para o processo.

- 1. **Cálculo do CVI**

O cálculo do CVI será realizado pelo índice de validade de conteúdo em nível de escala com base no método de concordância universal (S-CVI/UA). A classificação de relevância deve ser recodificada como 1 (escala de pontuação 3 ou 4) ou 0 (escala de pontuação 1 ou 2). A pontuação de concordância universal (UA) é dada como 1 quando o item atinge 100% de concordância dos especialistas (somente respostas 3 e 4 para todos os itens), caso contrário, a pontuação de UA é dada como 0. O S-CVI/UA é igual a soma dos escores de UA divididos pelo número de itens.

Soma dos escores de UA

S-CVI/UA = ______________________

Número de ítens

O valor de CVI será considerado aceitável para valores maiores que 0,83^7^.

**CRONOGRAMA GERAL**

| ***Mês/Ano →***  ***Atividades*** | 02/24 | 03/24 | 04/24 | 05/24 | 06/24 | 07/24 | 08/24 | 09/24 | 10/24 | 11/24 | 12/24 |
| --- | --- | --- | --- | --- | --- | --- | --- | --- | --- | --- | --- |
| **Encaminhar para Comitê de Ética** |  | X |  |  |  |  |  |  |  |  |  |
| **Revisão de Literatura** | X |  |  |  |  |  |  |  |  |  |  |
| **Escrever Material e Método** | X |  |  |  |  |  |  |  |  |  |  |
| **Coleta das fotografias** |  |  | X | X | X |  |  |  |  |  |  |
| **Apresentação aos especialistas** |  |  |  |  |  | X |  |  |  |  |  |
| **Análise dos Dados** |  |  |  |  |  |  | X |  |  |  |  |
| **Validação do índice** |  |  |  |  |  |  |  | X |  |  |  |
| **Escrever Resultados** |  |  |  |  |  |  |  |  | X |  |  |
| **Escrever Discussão e Conclusão** |  |  |  |  |  |  |  |  |  | X |  |
| **Encaminhar Artigo para Publicação** |  |  |  |  |  |  |  |  |  |  | X |

**O estudo iniciará após aprovação inicial do Comitê de Ética**

Referência:

1. Martignon S, Bartlett D, Manton DJ, Martinez-Mier EA, Splieth C, Avila V. Epidemiology of Erosive Tooth Wear, Dental Fluorosis and Molar Incisor Hypomineralization in the American Continent. Caries Res. 2021;55(1):1-11. doi: 10.1159/000512483. Epub 2021 Jan 13. PMID: 33440378.
2. Taji S, Seow WK. A literature review of dental erosion in children. Aust Dent J. 2010 Dec;55(4):358-67; quiz 475. doi: 10.1111/j.1834-7819.2010.01255.x. PMID: 21133936
3. Loomans B, Opdam N, Attin T, Bartlett D, Edelhoff D, Frankenberger R, Benic G, Ramseyer S, Wetselaar P, Sterenborg B, Hickel R, Pallesen U, Mehta S, Banerji S, Lussi A, Wilson N. Severe Tooth Wear: European Consensus Statement  on Management Guidelines. J Adhes Dent. 2017;19(2):111-119. doi: 10.3290/j.jad.a38102. PMID: 28439579.
4. Martignon S, Bartlett D, Manton DJ, Martinez-Mier EA, Splieth C, Avila V. Epidemiology of Erosive Tooth Wear, Dental Fluorosis and Molar Incisor Hypomineralization in the American Continent. Caries Res. 2021;55(1):1-11. doi: 10.1159/000512483. Epub 2021 Jan 13. PMID: 33440378.
5. Bartlett D, Ganss C, Lussi A. Basic Erosive Wear Examination (BEWE): a new scoring system for scientific and clinical needs. Clin Oral In- vestig. 2008 Mar;12(S1 Suppl 1):S65–8.
6. Yusoff MSB. ABC of content validation and content validity index calculation. Education in Medicine Journal. 2019;11(2):49–54. https://doi.org/10.21315/eimj2019.11.2.6
7. Lynn MR. Determination and quantification of content validity. Nursing Research. 1986;35(6):381–5.
