# Supplementary material for: Development and content validation of the Childhood Early Oral Aging Syndrome (CEOAS) index for the deciduous dentition: Research protocol
Source: PLoS One. 2024 Oct 25;19(10):e0310543. doi: 10.1371/journal.pone.0310543 (PMC11508467; doi:10.1371/journal.pone.0310543)
Supplement: S3 File — (DOCX) [file pone.0310543.s003.docx]

**Development and content validation of the Childhood Early Oral Aging Syndrome (CEOAS) index for the deciduous dentition**

**Researchers Responsible for the project:**

Amanda Rafaelly Honório Mandetta

São Paulo

2024

**Abstract**

Premature, non-physiological tooth wear in childhood has numerous repercussions for oral health. This is a growing problem with multifactorial causes and associated with the current lifestyle. The aim of the present study was the development and determination of content validity of the Childhood Early Oral Aging Syndrome (CEOAS) index for the primary dentition as a diagnostic and epidemiological survey tool considering the current changes found in this population.

**Introduction**

The increase in early and non-physiological dental wear found in the pediatric age, with numerous repercussions on oral health, is a growing reality, associated with multifactorial causes and the current lifestyle. With deeper knowledge about caries disease and its control, new demands arise, with the need for recognition among health professionals, aiming at prevention, disease control, and etiological factors^1^.

Non-carious diseases cause progressive and irreversible loss of dental structure, with the initial process associated with various causes without bacterial involvement^2^.

Dental wear is an age-related phenomenon. Erosive, attrition, and abrasion challenges throughout life will result in varying degrees of structural loss. It is difficult to define and quantify pathological wear, but the term pathological has been used to describe unacceptable levels of progressive wear, which can result in sensitivity, aesthetic impairment, and functional problems^3^.

The increase in the global prevalence of non-carious lesions reflects the need for changes in behavior, health education, epidemiological surveys, training, and calibrations, as well as a transdisciplinary perspective^1^.

Non-carious dental conditions, such as erosive dental wear, developmental enamel defects (DDE), including Molar Incisor Hypomineralization (MIH), Hypomineralization of Second Primary Molars (HSPM), and dental fluorosis are subjects of extensive research and growing concern for dentists worldwide^4^.

Epidemiological studies, with two recent systematic reviews, attempted to assess the prevalence of erosive wear in children and adolescents; however, comparison between studies was challenging since different indices were used to assess the severity of wear^4^.

The Basic Erosive Wear Examination (BEWE) index was developed as part of a consensus meeting in 2008 and is currently one of the most used indices in clinical and epidemiological studies^5^. However, it only considers erosive etiology as a diagnosis. Although erosion is considered the main cause of dental wear in children, other factors may contribute synergistically, such as attrition and abrasion^2^.

Hypomineralized enamel defects are highly prevalent, and due to the fragility of altered enamel, they are more susceptible to wear and early loss, thus contributing to the acceleration of premature aging^4^. The most used indices for fluorosis are Dean and Thylstrup and Fejerskov. The indices used for MIH and HSPM are modified DDE and the EAPD index^1^.

Currently, the absence of an index that contemplates the main factors determining the syndrome of early childhood aging and evaluates possible associations that coexist and imply the control and treatment of the disease is necessary and indispensable.

Therefore, this study aims to develop and validate the content of an index on the Syndrome of Early Childhood Oral Aging for deciduous dentition, as a diagnostic and epidemiological survey tool, considering the current alterations found in the pediatric population.

**Materials and methods**

**Study design**

For better understanding, the methodology will be divided into two stages, with the first stage directed towards the development of the index and the validation of the content.

1. Development of the index
2. Content validation by experts

2.1 Preparation of the content validation form

2.2 Selection of expert reviewers

2.3 Conducting content validation

2.4 Item review

2.5 Providing scores for each item

2.6 Calculation of the CVI

1. **Development of the SEPBI Index**

The Early Childhood Oral Aging Syndrome Index aims to evaluate clinical signs and symptoms related to early loss of dental structure associated with the most prevalent enamel defects today, which significantly contribute to oral aging. The clinical management of wear is also addressed in SEPBI scores 1, 2, and 3. Currently, the indices used for non-carious lesions do not concurrently address enamel defects, making this index innovative and extremely important for epidemiological surveys. Understanding the prevalence of factors that act independently or synergistically to accelerate the process of early aging is useful for new clinical treatment strategies.

The Early Childhood Oral Aging Syndrome Index (CEOAS) uses scores from 0 to 3 to assess dental wear and dental management, and scores I, II, and III, which should be used concurrently in cases of the presence of enamel defects, as shown in Table 1:

| **Table 1: Childhood Early Oral Aging Syndrome (CEOAS) index** | |
| --- | --- |
| **CEOAS 0** | **Absence of signs** |
| **CEOAS 1** | **Mild:** Presence of clinical signs in reversible stages, without sensitivity. Such cases require follow-up. |
| **CEOAS 2** | **Moderate:** Presence of advanced signs with sensitivity and compromised function. Such cases require restorative treatment and management of the sensitivity. |
| **CEOAS 3** | **Severe:** Presence of severe signs with pulp involvement and the risk of the loss of the tooth. Such cases require more invasive and rehabilitative treatment. |
| **CEOAS I** | **Presence of PSMH – Demarcated opacities** |
| **CEOAS II** | **Presence of PSMH – Post-eruptive breakdown (PEB)** |
| **CEOAS III** | **Presence of fluorosis** |

**CEOAS 0:** Absence of signs and symptoms of CEOAS.

**CEOAS 1**: Clinical findings compatible with chronological age and oral age. First clinical signs (facets with initial wear on enamel level without altering function), without symptoms. Such cases require clinical follow-up.

Due to the lack of studies on the physiological wear pattern in the primary dentition, slight tooth wear without symptoms, functional or esthetic problems compatible with physiological wear is considered in this score.

**CEOAS 2**: Signs of wear not compatible with chronological age (facets with deep wear, with dentin exposure and compromised function) and symptoms of hypersensitivity. May have gingival recession. Such cases require restorative treatment and management of the sensitivity. Due to the lack of studies on the physiological wear pattern in the primary dentition, atypical tooth wear for the age of the patient (pathological wear), with symptoms as well as functional and esthetic problems are considered in this score.

**CEOAS 3**: Signs of severe wear not compatible with chronological age, with pulp involvement (inflammation or necrosis), compromising function and the stomatognathic system. May have tooth fissures, root fissures, tooth fractures, gingival recession and changes in the temporomandibular joint. Loss of the tooth may occur. Such cases require invasive treatment (endodontic, restorative, rehabilitative or extractive).

**CEOAS I:** In the presence of primary second molar hypomineralization (PSMH) with demarcated opacities and without post-eruptive breakdown (PEB), the CEOAS I score should be recorded concomitantly with the 1, 2 or 3 score detected in the clinical examination.

**CEOAS II:** In the presence of primary second molar hypomineralization (PSMH) with post-eruptive breakdown (PEB), the CEOAS II score should be recorded concomitantly with the 1, 2 or 3 score detected in the clinical examination.

**CEOAS III:** In the presence of dental fluorosis, the CEOAS III score should be recorded concomitantly with the 1, 2 or 3 score detected in the clinical examination.

with the score 0,1,2 or 3 detected in the clinical examination.

The CEOAS index can be used in the simplified version, with the highest severity score found. In the full version, the SEPBI can be used followed by an odontogram , and each score will be assigned to a tooth (Figure 1).

| **Figure 1: Odontogram for the Infant Oral Premature Aging Syndrome (SEPBI) index** | | | | | | | | | | |
| --- | --- | --- | --- | --- | --- | --- | --- | --- | --- | --- |
| Tooth | 55 | 54 | 53 | 52 | 51 | 61 | 62 | 63 | 64 | 65 |
|  |  |  |  |  |  |  |  |  |  |  |
| Tooth | 85 | 84 | 83 | 82 | 81 | 71 | 72 | 73 | 74 | 75 |
|  |  |  |  |  |  |  |  |  |  |  |

1. **Content validation by experts**

**2.1 Preparation of the content validation form**

| **Content validity of Childhood Early Oral Aging Syndrome (CEOAS) index.**  Dear specialists,  This index was developed to provide a reliable, standardized tool for the investigation of clinical signs and symptoms related to early oral aging in childhood, with the inclusion of enamel defects, which are currently highly prevalent. The main purpose of the index is to serve as a tool for epidemiological surveys and enable knowledge on the prevalence and severity of tooth wear in the pediatric population, contributing to the planning of health actions based on the data collected.  We need your specialized opinion on the degree of relevance of each item to the domains measured. Your review should be based on the relevance, clarity and applicability of the score developed for the CEOAS index, as found in Table 2. We would be grateful for verbal or written suggestions that enable us to improve the content of the index. |
| --- |

| **Table 2: Items and assessment and validation criteria of the CEOAS index** | | | | |
| --- | --- | --- | --- | --- |
| Is the CEOAS index relevant to the diagnosis of early oral aging syndrome? | 1 | 2 | 3 | 4 |
|  | It is not relevant. | Relevant, but needs major revision. | Relevant, but needs minor revision. | Very relevant |
| Is the CEOAS index clear for the diagnosis of early oral aging syndrome? | 1 | 2 | 3 | 4 |
|  | It is not clear | Clear, but needs major revision. | Clear, but needs minor revision. | Very Clear |
| Is the CEOAS index relevant with regards to communication among health professionals and researchers? | 1 | 2 | 3 | 4 |
|  | It is not relevant. | Relevant, but needs major revision. | Relevant, but needs minor revision. | Very relevant |
| Is the CEOAS index applicable for diagnoses and epidemiological surveys? | 1 | 2 | 3 | 4 |
|  | It is not applicable. | Applicable, but needs major revision. | Applicable, but needs minor revision. | Very applicable |

**2.2 Selection of expert reviewers**

The selection of specialists for the assessment will be based on the individual experience of the specialist in the subject addressed in this study. Following recommendations for content validation, at least six specialists will be selected ^6^.

- 1. **Performing content validation**

Content validation will be carried out in person. The online content validation form will be sent to the experts with the items and criteria that should be evaluated (Table 1). A deadline for the evaluation will be requested and the entire process will be monitored. The images used to validate the Infant Oral Premature Aging Syndrome index for the primary dentition will be collected at the Clinic of the Faculty of Dentistry of the Metropolitan University of Santos (UNIMES) in the Children's Clinic discipline and in the Pediatric Dentistry Specialization. After consent from parents or guardians, children will also be informed, in appropriate language, about the objectives and procedures of the research and must agree to voluntarily participate in it, expressing their assent, which will be recorded in the Free and Informed Assent Form.

**2.4 Item review**

During the validation process, experts will be asked to critically review the content before scoring. Verbal and written suggestions for improving the content will be encouraged.

- 1. **Provide scores for each item**

After the review, experts must score their assessment of the relevance, clarity and applicability of the content for each item (Table 2) and deliver them within the deadline provided for the process.

- 1. **CVI calculation**

The CVI calculation will be performed using the content validity index at scale level based on the universal agreement method (S-CVI/UA). The relevance rating should be recoded as 1 (scoring scale 3 or 4) or 0 (scoring scale 1 or 2). The universal agreement (UA) score is given as 1 when the item reaches 100% expert agreement (only responses 3 and 4 for all items), otherwise the UA score is given as 0. The S-CVI /UA is equal to the sum of the UA scores divided by the number of items.

Sum of UA scores

S-CVI/UA = ______________________

Number of items

The CVI value will be considered acceptable for values greater than 0.83^7^ .

**GENERAL SCHEDULE**

| ***Month/Year →***  ***Activities*** | 02/24 | 03/24 | 04/24 | 05/24 | 06/24 | 07/24 | 08/24 | 09/24 | 10/24 | 11/24 | 12/24 |
| --- | --- | --- | --- | --- | --- | --- | --- | --- | --- | --- | --- |
| **Forward to Ethics Committee** |  | X |  |  |  |  |  |  |  |  |  |
| **Literature review** | X |  |  |  |  |  |  |  |  |  |  |
| **Writing Material and Method** | X |  |  |  |  |  |  |  |  |  |  |
| **Collection of photographs** |  |  | X | X | X |  |  |  |  |  |  |
| **Presentation to experts** |  |  |  |  |  | X |  |  |  |  |  |
| **Data analysis** |  |  |  |  |  |  | X |  |  |  |  |
| **Index validation** |  |  |  |  |  |  |  | X |  |  |  |
| **Write Results** |  |  |  |  |  |  |  |  | X |  |  |
| **Write Discussion and Conclusion** |  |  |  |  |  |  |  |  |  | X |  |
| **Submit Article for Publication** |  |  |  |  |  |  |  |  |  |  | X |

**The study will begin after initial approval from the Ethics Committee**

Reference:

1. Martignon S, Bartlett D, Manton DJ, Martinez- Mier EA, Splieth C, Avila V. Epidemiology of Erosive Tooth Wear, Dental Fluorosis and Molar Incisor Hypomineralization in the American Continent. Caries Res. 2021;55(1):1-11. doi : 10.1159/000512483. Epub 2021 Jan 13. PMID: 33440378.
2. Taji S, Seow WK. A literature review of dental erosion in children. Australia Dent J. 2010 Dec;55(4):358-67; quiz 475. doi : 10.1111/j.1834-7819.2010.01255.x. PMID: 21133936
3. Loomans B, Opdam N, Attin T, Bartlett D, Edelhoff D, Frankenberger R, Benic G, Ramseyer S, Wetselaar P, Sterenborg B, Hickel R, Pallesen U, Mehta S, Banerji S, Lussi A, Wilson N. Severe Tooth Wear: European Consensus Statement on Management Guidelines. J Adhes Dent . 2017;19(2):111-119. doi : 10.3290/j.jad.a38102. PMID: 28439579.
4. Martignon S, Bartlett D, Manton DJ, Martinez- Mier EA, Splieth C, Avila V. Epidemiology of Erosive Tooth Wear, Dental Fluorosis and Molar Incisor Hypomineralization in the American Continent. Caries Res. 2021;55(1):1-11. doi : 10.1159/000512483. Epub 2021 Jan 13. PMID: 33440378.
5. Bartlett D, Ganss C, Lussi A. Basic Erosive Wear Examination (BEWE): a new scoring system for scientific and clinical needs. Clin Oral Investig . 2008 Mar;12(S1 Suppl 1):S65–8.
6. Yusoff MSB. ABC of content validation and content validity index calculation. Education in Medicine Journal . 2019;11(2):49–54. https://doi.org/10.21315/eimj2019.11.2.6
7. Lynn MR. Determination and quantification of content validity. Nursing Research. 1986;35(6):381–5.
